# Interface shear behavior of a shaft lining concrete-high density polyethylene material under different pre-imposed normal stresses

**DOI:** 10.1371/journal.pone.0264691

**Published:** 2022-03-17

**Authors:** Tao Zhang, Chi Zhang, Jiuqun Zou

**Affiliations:** 1 School of Transportation and Civil Engineering, Nantong University, Nantong, China; 2 State Key Laboratory for Geomechanics and Deep Underground Engineering, China University of Mining and Technology, Xuzhou, China; 3 School of Civil Engineering and Architecture, Anhui University of Science and Technology, Huainan, China; University of Vigo, SPAIN

## Abstract

As an important part of a double-layer shaft lining in China, a polyethylene sandwich can reduce or even eliminate the constraint effect on the inner shaft lining, which is confined by the outer shaft lining, thus improving the integrity of the inner shaft lining concrete. To reveal the interface shear mechanism of the concrete-high density polyethylene (HDPE) material, a series of 16 direct shear tests were performed under high normal loads (0.6~4 MPa) with four different surface morphologies. The experimental results showed that the interfaces exhibit a clear strain-softening property during shear, and the shear strength increases linearly with increasing normal stress. Three shearing mechanisms, plowing, localized plastic deformation, and dilatancy, were observed in these experiments. The research results are of great theoretical and practical value for understanding the shear mechanism and predicting the shear strength of a shaft lining concrete-HDPE interface under high normal stress.

## Introduction

**Fig 1:**
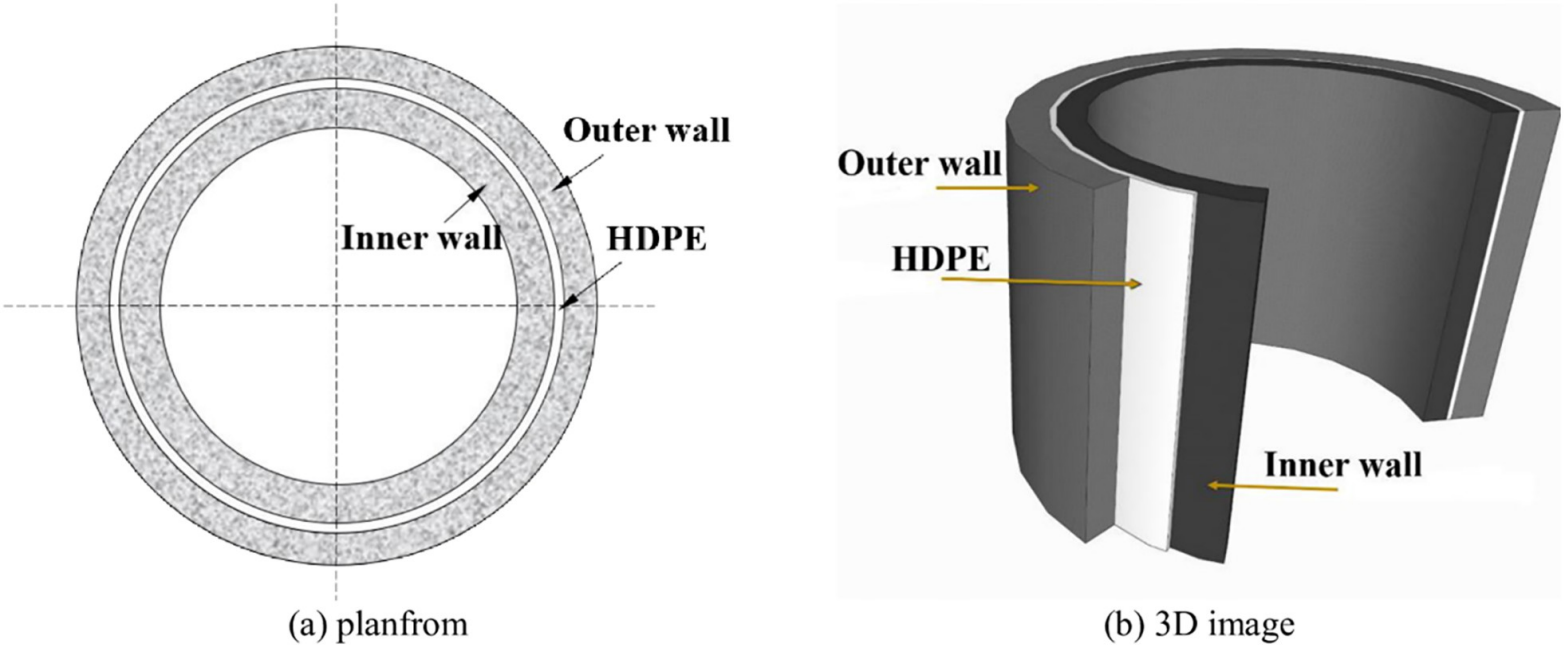
Schematic plot of the composite vertical shaft lining. (a) planfrom. (b) 3D image.

**Fig 2:**
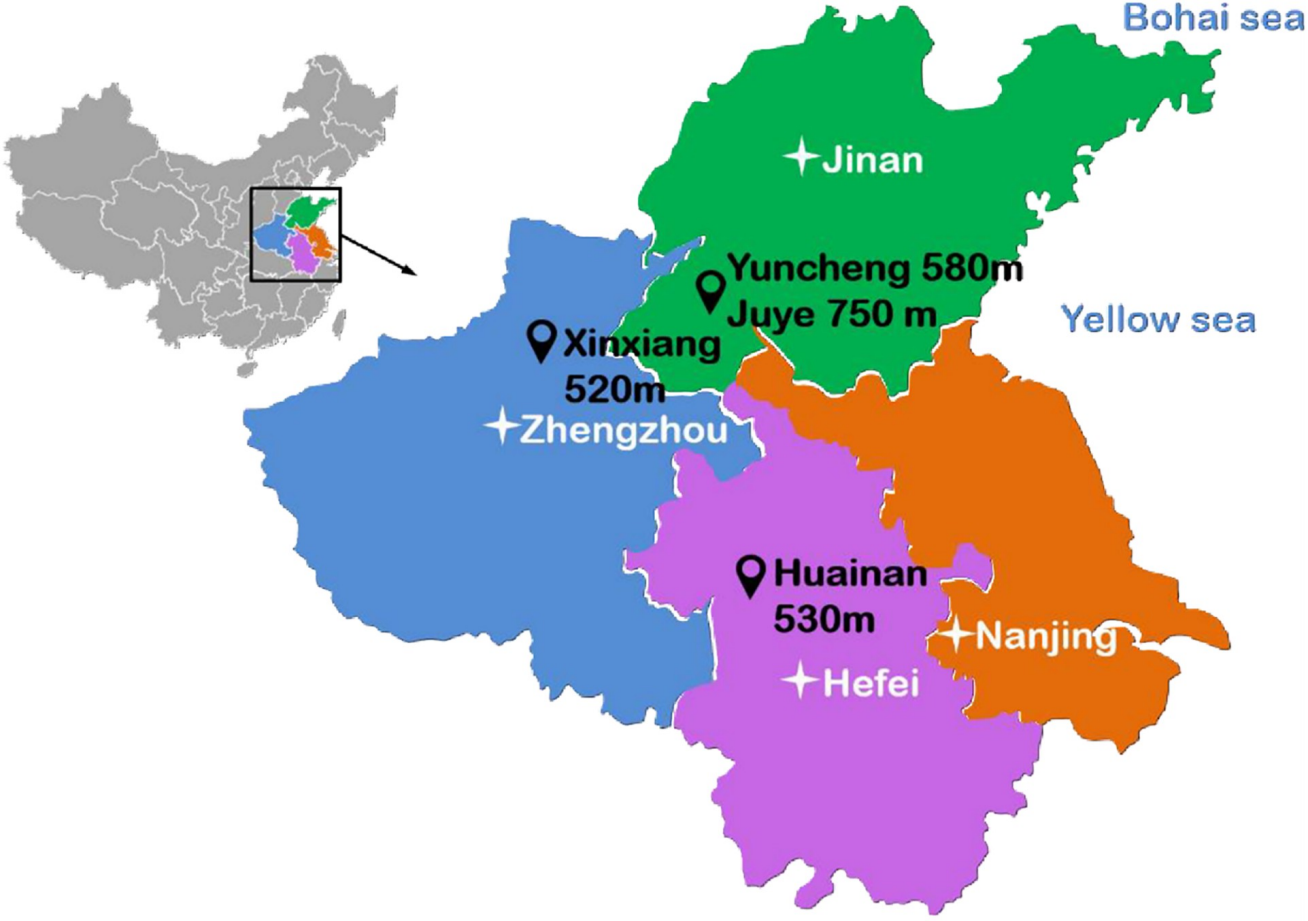
Some shaft linings with soil depths exceeding 500 m in the eastern region of China.

A composite vertical shaft lining with concrete and a sandwiched high-density polyethylene is one of China’s widely used types of shaft structures in mining engineering, as shown in Fig 1. To date, more than 30 vertical shaft linings have been constructed in the deep alluvium exceeding 500 m. Fig 2 shows some vertical shaft linings with soil depths exceeding 500 m in the eastern region of China. As a buffer and isolation layer resisting high pore water pressure and vertical additional stress induced by stratum settlement, the sandwiched HDPE plays a vital role in improving the integrality of the inner wall [1, 2]. At present, the use of the HDPE depends solely on experience in the design and construction, which did not consider the effects of horizontal geostress and interface roughness on the interaction between the concrete and HDPE. Thus, it is essential to understand the fundamental physics of the interaction at the concrete-HDPE interface to develop the precise design methods and structure-HDPE interaction models. Various studies have been performed to study the mechanical properties of HDPE materials, with the majority focusing on welding technology, paving technology, and physical or chemical damage [3–9]. Several previous studies have focused on the shear behaviors of the interfaces between civil engineering materials and HDPEs [10–19]. Vangla and Gali [11] conducted large scale shear tests on the interface between a smooth geomembrane and sands with varying particle sizes and morphology at different normal stresses, the results showed that morphology of the sands have major influence on the interface shear strength. The results of direct shear tests at the sand-geosynthetic interface conducted by Punetha et al. [12] revealed that the shearing mechanism includes interlocking and fiber stretching while sliding, indentation and plowing for sand-geomembrane interface. Cen et al. [16] investigated the cyclic shear behavior of geomembrane-concrete interface and observed that the textured geomembrane-concrete interface presents higher cyclic friction angles. Anubhav and Basudhar [19] proposed a non-linear constitutive model for predicting the pre-peak and the post-peak mechanical behavior of the soil-geotextile interface, and the peak interface shear strength was found to be significantly higher for the coarse textured geotextile. However, specific studies on the shear mechanisms of concrete-HDPE interfaces under high normal stress are limited.

The shear strength mainly depends on the external load, joint roughness, shear rate, material property, and bonding state [11–16, 19–22]. The surface morphology between HDPE and another material is an important factor in determining the integrality and service life of HDPE materials [23]. Surface morphology has gained significant attention through laboratory experiments, largely on joint surfaces comprised of different materials (e.g., concrete-rock joints, rock joints, or cemented-rock joints) [24–28]. In actual projects, concrete-HDPE interfaces under high pressures are rough and dilative because the concrete surface is not smooth but instead exhibits asperities due to rapid and complex construction conditions.

**Fig 3:**
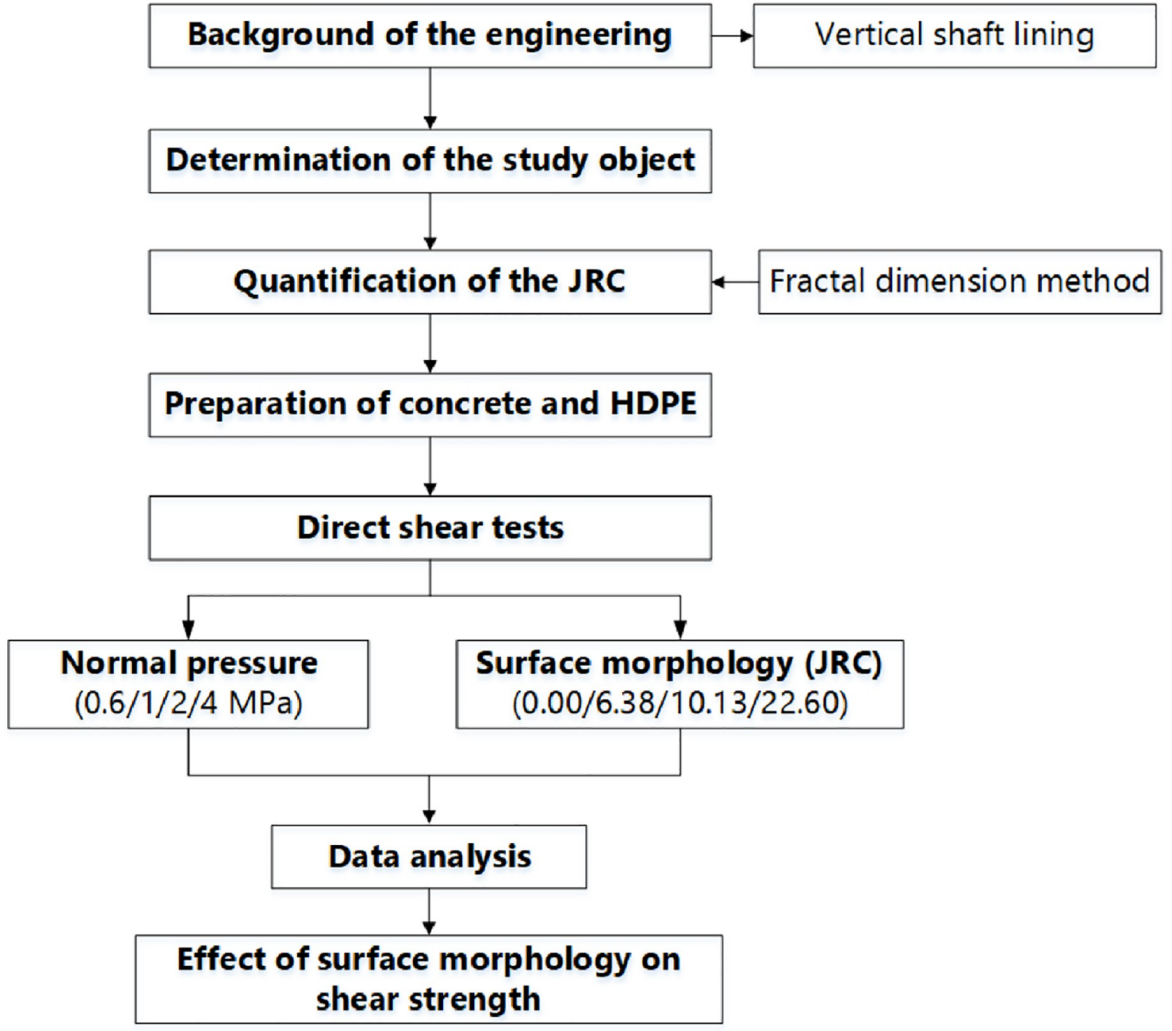
Flow chart of the experiment.

Not many studies are available on the effect of surface morphology on concrete-HDPE interface. The main objective of this paper was to conduct a laboratory experiment on the shear behavior of concrete-HDPE interfaces using a high-pressure direct shear apparatus. Direct shear tests were performed on four interface profiles with different joint roughness coefficients (JRCs), and the effect of the JRC on shear strength was investigated. The results of this study will contribute to a more reasonable design of the composite shaft lining. Fig 3 shows the flow chart of the experiment.

## Laboratory investigation

### Quantification of the JRC

Traditionally, as an important geometrical parameter of joint roughness, the JRC directly affects the friction angle, dilatancy, and peak shear strength, which is considered to be generated from local protruding and deflected planes at both small and large scales. Barton [29] first introduced the concept of the JRC. Barton and Choubey [30] performed direct shear tests on joint rock samples and proposed ten standard roughness profiles with numerical representations from ‘0’ to ‘20’. Each roughness profile represents a specific roughness coefficient, where a value of ‘0’ corresponds to the smoothest profile, and a value of ‘20’ corresponds to the roughest profile. Nevertheless, the evaluation of the JRC through profile approximation is empirical and subjective. To increase the accuracy of the JRC, several researchers have explored statistical techniques to determinate the JRC values from profiles (e.g., ultimate slope of the profile (*λ*), mean deviation roughness index (*R*_*a*_), root mean square roughness index (*R*_*q*_), a standard deviation of the angle (*σ*_*i*_), mean square value roughness index (*M*_*s*_), root mean square of the first deviation of the profile *(Z*_*2*_) and fractal dimension (*D*)) [31–34]. In this study, four sawtooth profiles were calculated with different convex angles at identical heights. The fractal dimension method was suited for quantifying the JRC due to its accuracy and widespread use.

**Fig 4:**
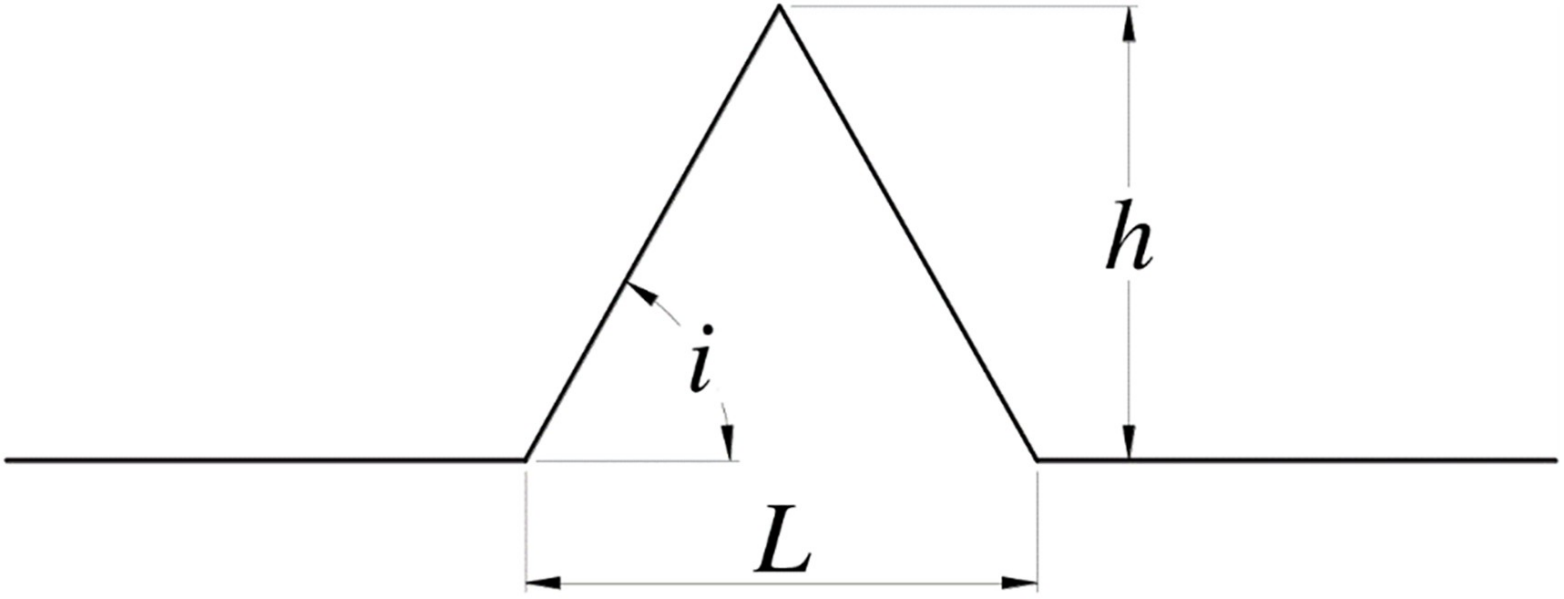
Generator of Koch’s original.

Xie and Pariseau [35] proposed that the joint profiles had similar structures, such as the Koch curve, based on which the traditional generator of Koch’s original postulates was popularized and applied to simulate natural random joints, as shown in Fig 4. Fig 4 shows that angle *i* of the generator can be varied from 0° (*h* = 0) to 90° (*L* = 0). The theoretical model can be expressed as

$$\left.\begin{array}{l} N=4\\ 1/r=2\left[1+\text{cos}\;tg^{-1}\left(2h/L\right)\right] \end{array}\right\}$$ (1)

where *N* is the number of evenly partitioned straight lines, *r* is the ratio of similarity, *h* is the height of the generator (i.e., the height of each sawtooth profile), and *L* is the base length of the generator.

Conveniently, the fractal dimension of the profiles can be calculated from the following equation [36]

$$D=\frac{\text{lg}\;N}{\text{lg}\;\left(1/r\right)}$$ (2)

where *D* is the fractal dimension.

Eq (2) can be transformed into Eq (3)

$$D=\frac{\text{lg}\;4}{\text{lg}\left[2\left(1+\text{cos}tg^{-1}2h/L\right)\right]}$$ (3)

**Fig 5:**
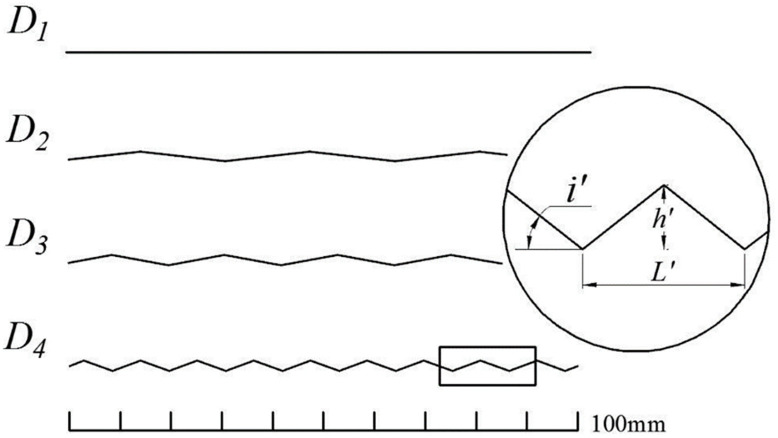
Design conditions and contour lines of samples D1-D4.

According to the aforementioned method, four joint profiles were used for the direct shear tests in this study, where group D1 corresponds to the smooth profile and groups D2, D3, and D4 are sawtooth profiles with different oblique angles, as shown in Fig 5. The geometrical parameters of the four samples are listed in Table 1. Similarly, angle *i’* of the generator can be varied from 0° (*h’* = 0) to 90° (*L’* = 0). The modified theoretical model has the following form

$$\left.\begin{array}{l} N=2\\ 1/r=2\;\text{cos}\;tg^{-1}\left(2h^{\prime }/L\prime \right) \end{array}\right\}$$ (4)

where *h’* and *L’* are the height and base length of the generator in this study, respectively.

**Table 1:** Geometrical parameters and JRC values of the joint profiles for samples D1-D4.

Group No.	Base length	Height	Angle	Fractal dimension	JRC
	*L’* (mm)	*h’* (mm)	*i’* (°)	*D*	
D1	-	0.0	0.0	1.0000	0.00
D2	25.00	1.5	6.8	1.0104	6.38
D3	16.67	1.5	10.2	1.0235	10.13
D4	8.33	1.5	19.8	1.0965	22.60

From Eqs (2) and (4), we have the following relation

$$D=\frac{\text{lg}\;2}{\text{lg}\left[2\text{cos}tg^{-1}\left(2h\prime /L\prime \right)\right]}$$ (5)

The relationship between the fractal dimension and the JRC value was proposed by Xie and Pariseau [35]

$$\text{JRC}=85.2671{\left(D-1\right)}^{0.5679}$$ (6)

From Eqs (5) and (6), the JRC value can be calculated as

$$\text{JRC}=85\text{.2671}{\left(\frac{\text{lg}\;2}{\text{lg}\left[2\text{cos}tg^{-1}\left(2h\prime /L\prime \right)\right]}-1\right)}^{0.5679}$$

### Determination of normal stress

In many engineering problems, asides from their good anti-seepage contributions, HDPE suffers from normal loads or overburden pressures. Most normal stress are 0.2–0.5 MPa [13, 21, 27]. In contrast, the normal load or horizontal stress on the composite shaft lining increases with an increasing depth of coal mining. Fig 2 shows some shaft linings with soil depths exceeding 500 m in the eastern region of China. Hoek and Brown [37] found that the vertical stress was closely linked to the exploited depth and density of the rock mass or soil mass. However, there is no unified approach for computing the horizontal stress in deep strata, and the results have yet to be proven using measured data in engineering tests [38, 39]. At present, the horizontal stress exerted on the shaft lining is determined as follows

$$\sigma_{h}=\gamma_{h}H$$ (7)

where *σ*_*h*_ is the horizontal stress, *γ*_*h*_ is the unit weight (typically 0.013 kN/m^3^ in the soil mass [38]), and *H* is the depth of the calculation point. Considering the high horizontal stress exerted on shaft linings, the normal stresses were fixed at 0.6, 1, 2, and 4 MPa for all samples.

## Test samples and apparatus

### Concrete samples

Concrete samples with dimensions of 100×80×30 mm and cubic compression strength of 60 MPa at 28 days were used in this study. The coarse aggregate was basalt-based gravel with a size of less than 8 mm, and the fine aggregate was common silica-based river sand. The water-reducing agent was selected from the Institute of Building Sciences in Nanjing, and the added quantity of water was 3%. PC52.5 cement was used, and the mix proportion of concrete was as follows: cement: water: fine aggregate: coarse aggregate = 1: 0.36: 1.10: 2.50, which is in comparison with some similar studies in Table 2. All samples were cast in specially designed molds with different profiles. The geometrical parameters of the four profiles are listed in Table 1.

**Table 2:** Mix proportion designed in this study and some previous studies.

Cement grade	Cement	Water	Fine aggregate	Coarse aggregate	Compression strength	Origin
	kg	kg	kg	kg	MPa	
PC52.5	471	169.5	518.8	1178.5	60.00	Herein
PC42.5	512	163.7	581.8	1093.7	61.00	[40]
PC53.0	450	130	638	1110	56.43	[41]
PC53.0	465	130.2	651	1088.1	64.38	[42]

### HDPE material

**Fig 6:**
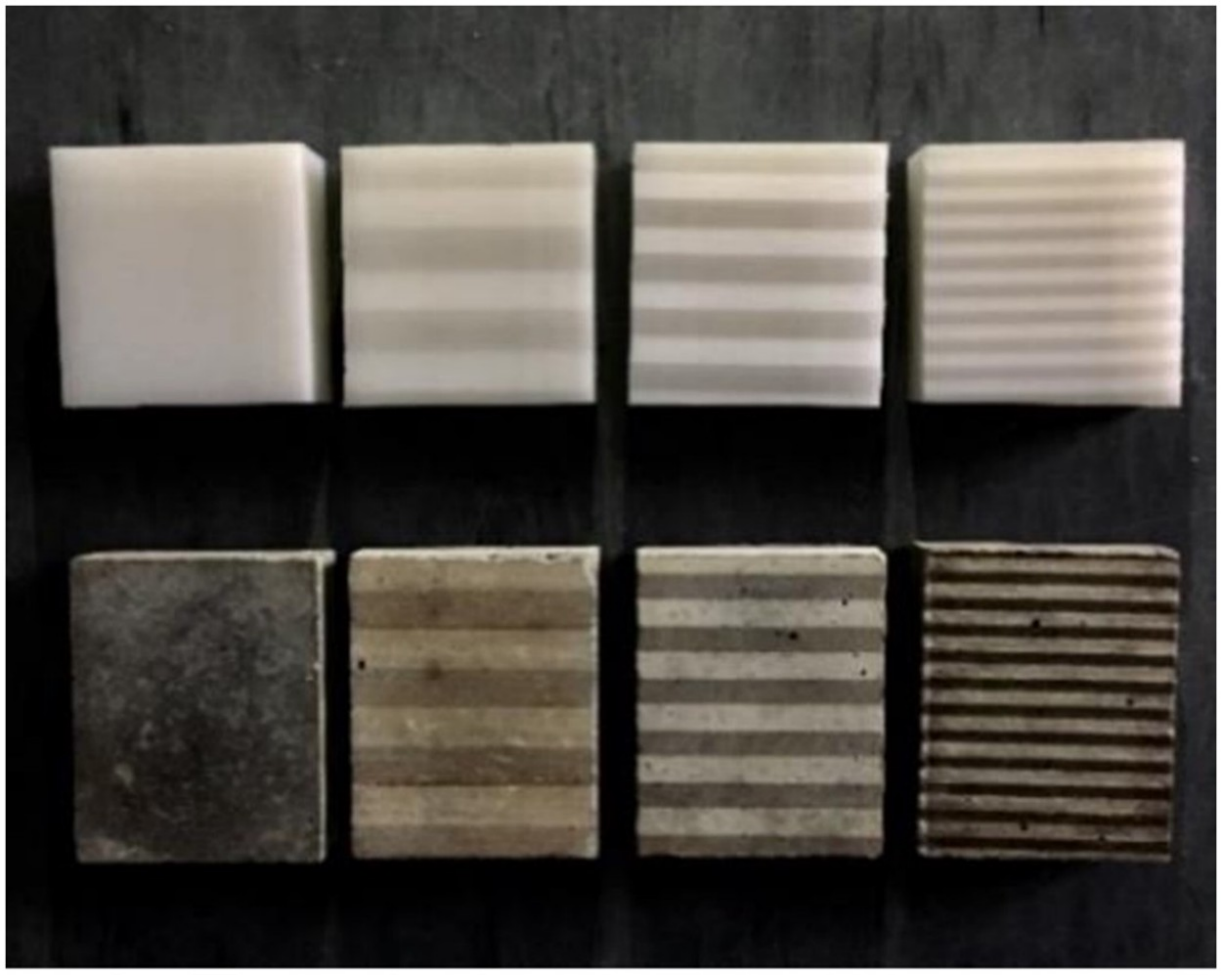
Image of test samples with different joint profiles.

A smooth HDPE that is commercially available and often used in engineering applications was used in this study. Samples with sawtooth p[rofiles were processed by high-pressure water-jet cutting to ensure their accuracy and integrality, as shown in Fig 6. Several key properties of this HDPE provided by the manufacturer are listed in Table 3.

**Table 3:** Test report of HDPE provided by the manufacturer.

Property	HDPE
Tensile yield strength (portrait/landscape), MPa	12.5/12.3
Flexural yield strength (portrait/landscape), MPa	6.5/7.3
Density, kg/m^3^	960

### Test setup and procedure

**Fig 7:**
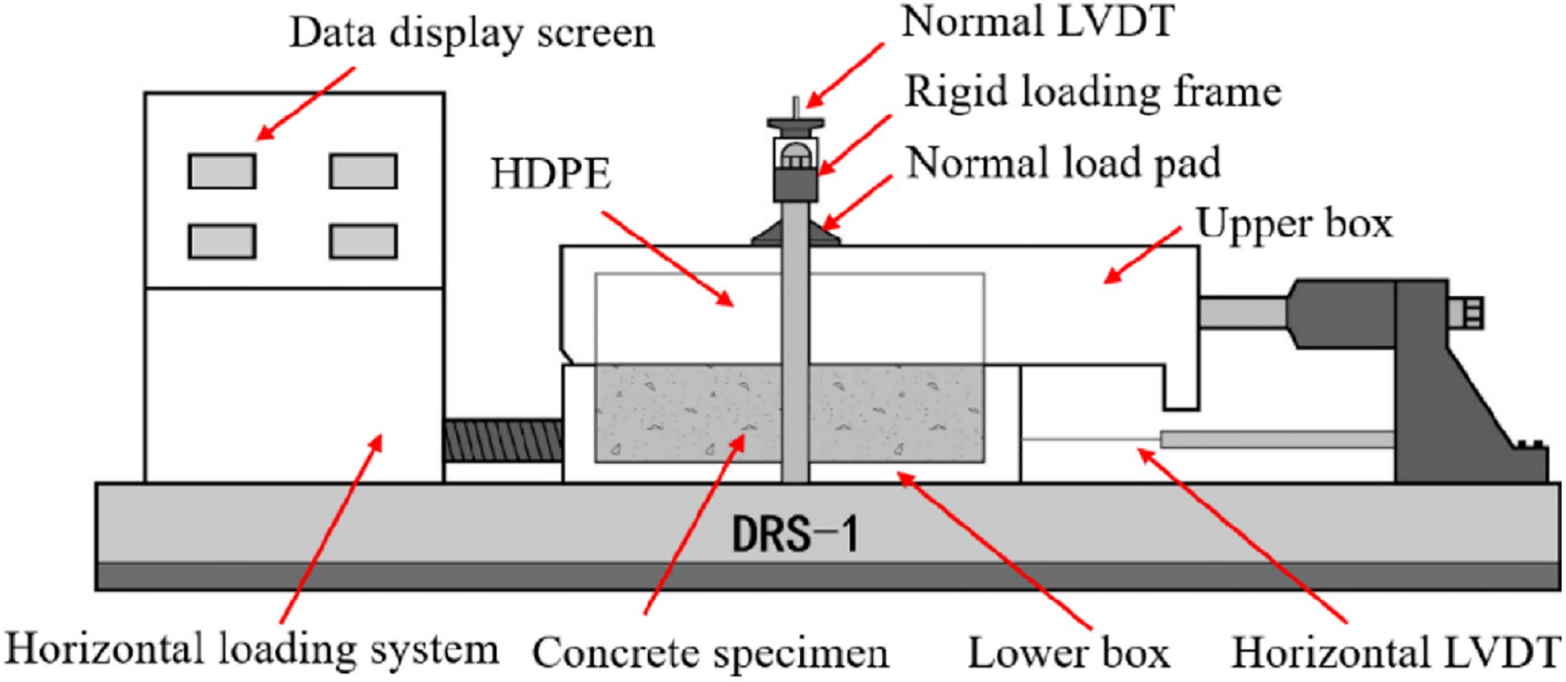
Schematic diagram of direct shear machine DRS-1.

The tests on concrete-HDPE interfaces were performed with a high-pressure direct shear machine, whose fixed upper half-box contained the HDPE specimen and whose moveable lower half-box contained the concrete specimen, as shown in Fig 7.

The normal load that acts on the upper box remained constant during the shearing process. Each shear test was conducted using a rate of moving deformation of 0.5 mm/min. Normal and shear loads were collected using a computerized data logging system, and the normal and shear displacements were monitored and saved by linear variable differential transducers (LVDTs). Each LVDT has a measurement range of 10 mm and a sensitivity of 0.005 mm. Each group was performed by applying normal stresses of 0.6, 1, 2, and 4 MPa, as listed in Table 4.

**Table 4:** Details and results of the direct shear tests.

Group No.	Normal Stress (MPa)	Shear Stress (MPa)		Peak Strength			Residual Strength		
		Peak	Residual	Friction Angle	Adhesion (MPa)	Coefficient *R*^2^	Friction Angle	Adhesion (MPa)	Coefficient *R*^2^
D1	0.6	0.296	0.222	23.83°	0.0015	0.9924	12.68°	0.0659	0.9947
	1	0.468	0.290						
	2	0.797	0.481						
	4	1.801	0.980						
D2	0.6	0.323	0.264	28.33°	0.0084	0.9986	24.14°	0.0424	0.9992
	1	0.527	0.425						
	2	1.132	0.840						
	4	2.148	1.580						
D3	0.6	0.350	0.334	29.50°	0.0082	0.9999	24.20°	0.0708	0.9998
	1	0.568	0.524						
	2	1.145	0.975						
	4	2.270	1.866						
D4	0.6	0.517	0.502	40.51°	0.0079	0.9991	33.33°	0.1636	0.9963
	1	0.901	0.831						
	2	1.664	1.560						
	4	3.442	2.760						

## Experimental results

The direct shear test results and analysis of the entire investigation are presented in this section. The test results are presented by plotting four types of graphs: the shear stress-shear displacement curves, vertical displacement-horizontal displacement curves, friction coefficient-normal stress curves, and shear strength envelopes.

### Effect of normal stress and JRC on the shear strength

**Fig 8:**
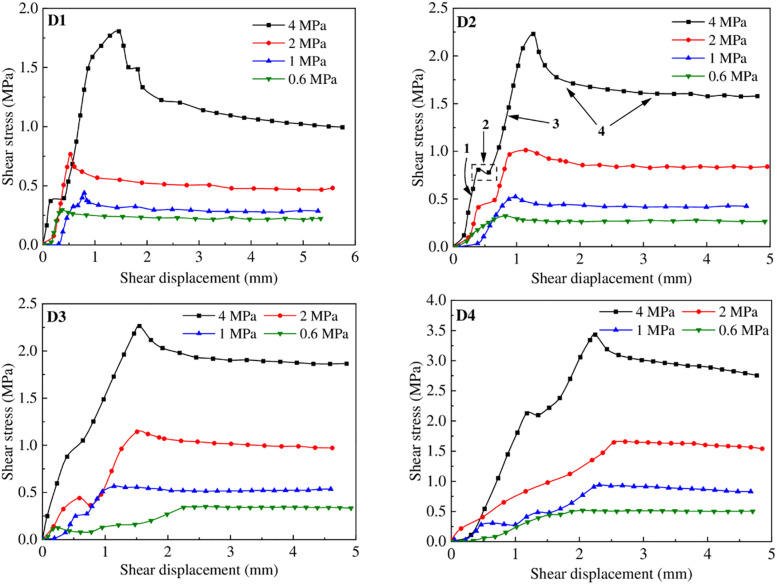
Shear stress versus shear displacement plots: (a) D1; (b) D2; (c) D3; and (d) D4.

Fig 8 shows the typical shear stress versus shear displacement or horizontal displacement relationships for all samples. In the case of samples D1 and D2, the interface shear stress gradually increased with shear displacement up to the peak stress. When shearing continued, there was a decrease in shear stress until a constant or residual value was attained. Furthermore, clear strain softening was observed, and higher normal stress resulted in a more discernible softening phenomenon. In contrast, no marked strain-softening was observed for samples D3 and D4 when the normal stress was less than 4 MPa. The residual state was attained in every sample under arbitrary normal stresses; for a given applied normal stress, the shear displacement corresponding to the peak shear strength increased with increases in the JRC. The present result is very similar to the interface behavior between sand and geomembrane [11, 12, 43], ballast-geosynthetic [44], geotextile and geomembrane [45].

The results also show a clear trend of increasing peak shear stress with increasing normal stress, which occurs due to the increasing contact area between the concrete and geomembrane interface with increasing normal stress. Similarly, as expected, higher residual shear stress occurred under higher normal stress.

**Fig 9:**
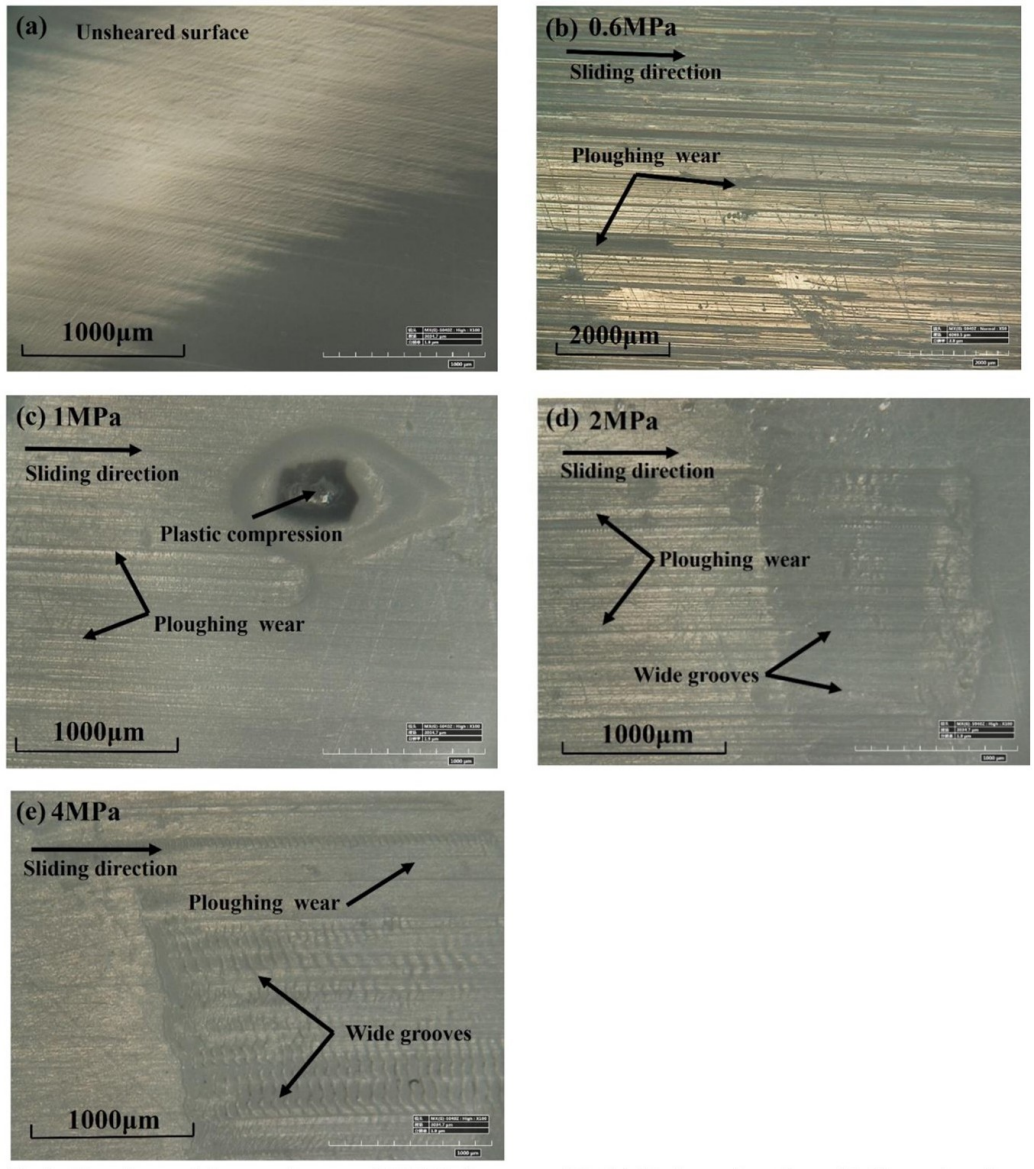
Abrasion and damage images of HDPEs for group D2: (a) Unsheared surface; (b) Sheared surface under normal stress of 0.6 MPa; (c) Sheared surface under normal stress of 1 MPa; (d) Sheared surface under normal stress of 2 MPa; (e) Sheared surface under normal stress of 4 MPa.

An integrated shear stress-shear displacement curve can accurately reflect the interface shear behaviors. Globally, most curves can be classified into four stages during complete shearing. For example, these stages are marked for group D2 when the normal stress is 4 MPa. In stage 1, the shear stress increases with increasing deformation until the first peak, which can be attributed to the local asperities between the concrete-HDPE surfaces. In stage 2, the shear stress decreases or slightly remains constant because of loss of local asperities during shearing. A similar phenomenon was also observed for granite joints by Singh and Basu [46]. After stage 2, the local lost asperities produce wear materials between the concrete and HDPE interfaces, which results in the second peak shear stress. Fig 9 presents the images for the HDPE samples before and after shearing under four different normal stresses for group D2. Fig 9(c) clearly shows that the lost asperities embed themselves into the HDPE surface, plowing the surface during shearing with increasing normal stress. For normal stress greater than 1 MPa, wide grooves are plowed by the saw-toothed asperities, which causes serious plastic deformation of the HDPE surfaces. The plowing of deeper trenches and wider grooves also requires higher shear stress at the concrete-HDPE interfaces, which results in high interface shear strength at high normal stress. In stage 4, post-peak shear strength decreases with increasing shear displacement, but the shear strength remains largely unchanged during the following shearing. This phenomenon can result from the breaking down of local asperities into finer particles or from most particles having embedded themselves into the HDPE surfaces. Similar observations of shear behavior were reported in other studies [11, 47]. The saw-toothed asperities of the concrete and HDPEs remain largely intact during shearing, which can be attributed to the high-strength concrete and elastic deformation of the HDPEs.

**Fig 10:**
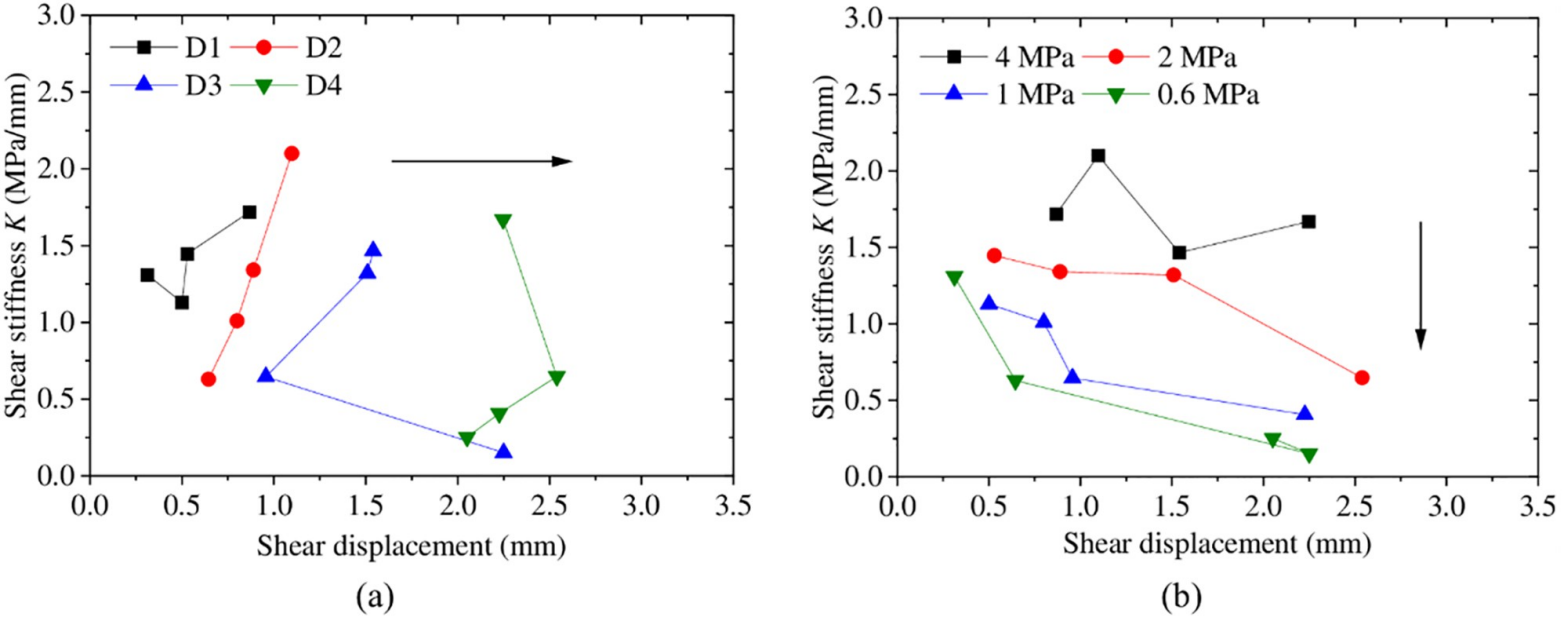
Plot of shear stiffness versus shear displacement (a) with different surface morphologies; (b) with different normal stresses.

Fig 10 shows the initial shear stiffness versus shear displacement for four groups. The shear stiffness is defined as the ratio of the shear stress before the peak to the corresponding shear displacement. It is obviously that the shear stiffness increases with increasing JRC, as shown in Fig 10(a). Also the effect of the stress level on the shear stiffness is more pronounced for smaller shear displacement, and the shear stiffness is much higher for higher normal stress, as shown in Fig 10(b).

### Effect of JRC on the friction angle

**Fig 11:**
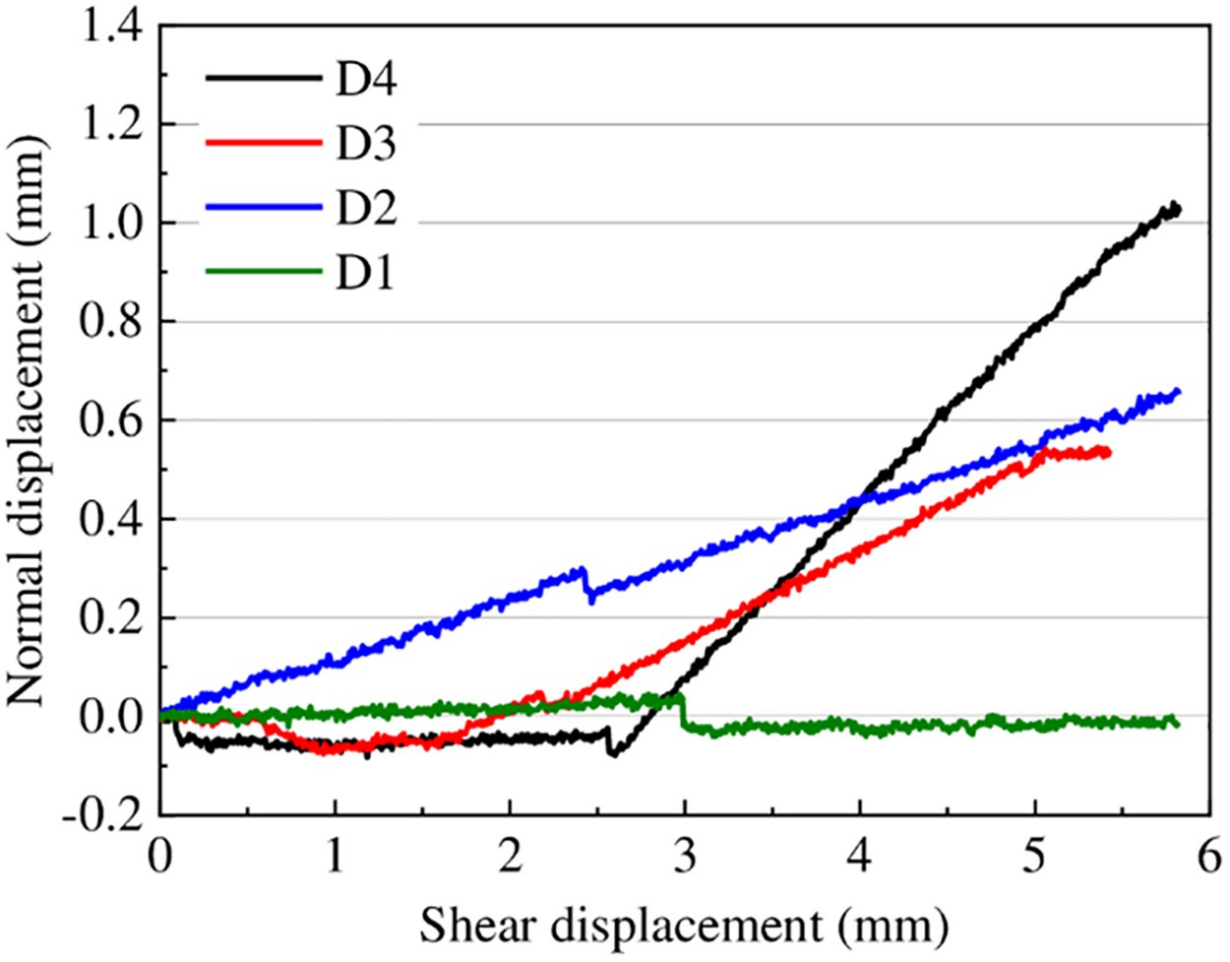
Normal displacement versus shear displacement of four joints under normal stress of 2 MPa.

Fig 11 illustrates the typical results of normal displacement versus shear displacement of four surface morphologies under normal stress of 2 MPa. The results indicate that the samples dilated with a constant dilation rate, which corresponds to their asperity base angles (0°, 6.8°, 10.2°, and 19.8°), which plays a significantly vital role in the determination of mechanical properties, particularly the shear strength [26, 48]. Patton [49] conducted direct shear tests on artificial plaster joints with a regular saw-toothed shape and obtained the envelope line of the peak strength, as shown in Eq (8).

$$\tau_{\text{p}}=\sigma_{\text{n}}\text{tan}(\phi_{\text{b}}+i)$$ (8)

where *τ*_p_ is the peak shear strength, *σ*_n_ is the normal stress, *ϕ*_b_ is the basic friction angle, and *i* is the asperity angle of the saw-toothed surface.

**Fig 12:**
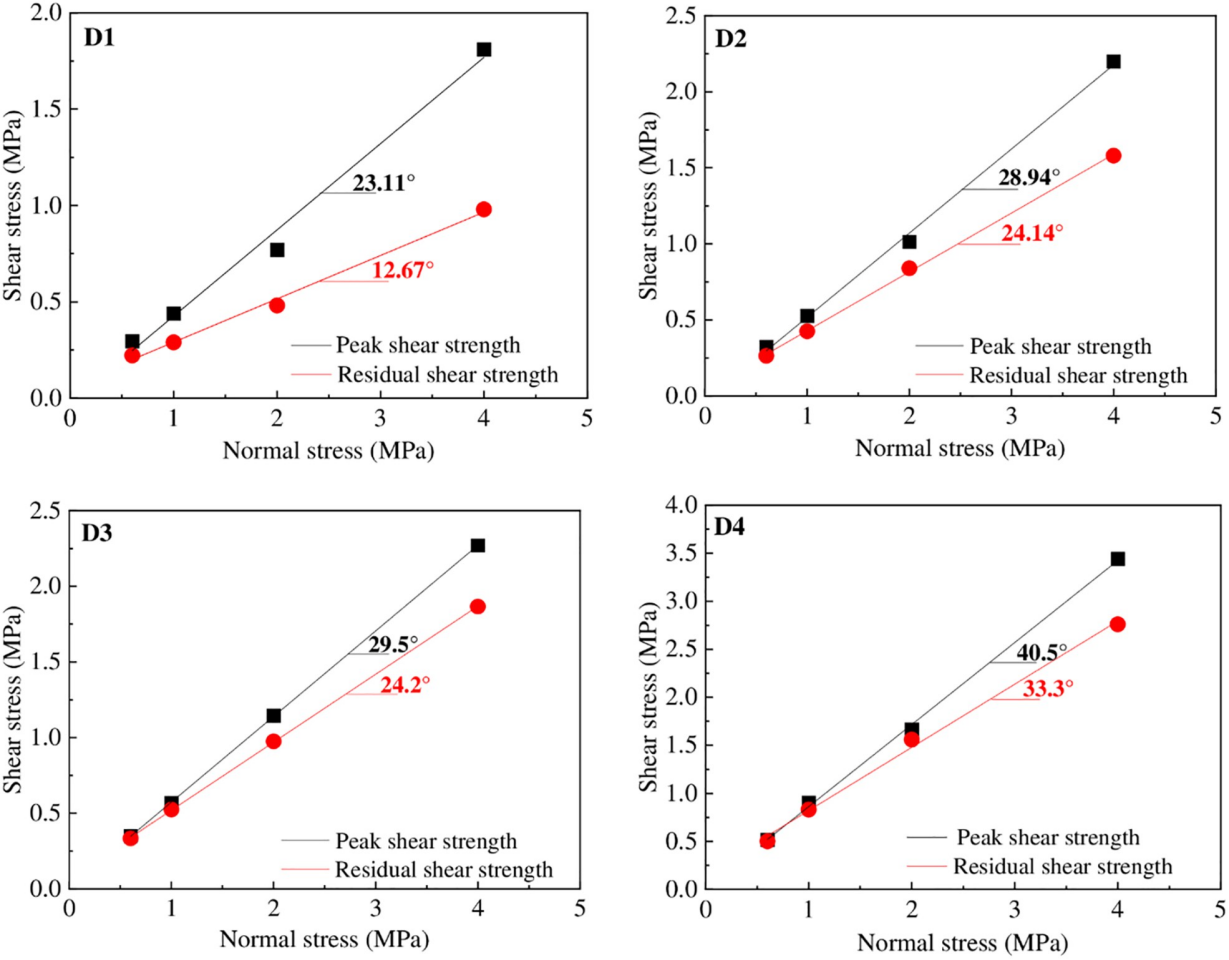
Peak and residual friction angles from plots of shear stress versus normal stress.

The peak and residual friction angles for groups D1-D4 were determined by plotting the peak and residual strength versus normal stress, and the strength envelopes were obtained by fitting linear regression lines through the data points, as shown in Fig 12. The peak strength envelopes have straight lines with regression coefficients greater than 0.989. All shear failure envelopes for the concrete-geomembrane structure can be described by the Mohr-Coulomb failure criterion, and the interface friction angle is obtained via Eq (9).

$$\tau =c+\sigma_{n}\text{tan}\phi$$ (9)

where *τ* is the interface shear stress, *σ*_*n*_ is the total normal stress, *ϕ* is the interface friction angle, and *c* is the adhesion and cohesion (which is negligible for unbonded contact).

Table 4 summarizes the shear parameters for all concrete-HDPE interfaces. As a result, the peak failure envelopes can be represented by friction angles of 23.83°, 28.33°, 29.50°, and 40.50°, which can be attributed to the increased roughness of the regular saw-shaped samples. The residual strengths were 12.68°, 24.14°, 24.20°, and 33.33°, which are typical post-peak strength losses for the concrete-HDPE interfaces tested in this study.

## Comparison and discussion

### Soil-HDPE interface

As in the aforementioned cases and to the authors’ knowledge, few experimental studies have been conducted on the behavior of concrete-HDPE interfaces. Fine sand (FS)-HDPE interfaces tested by Vangla and Gali [11] resulted in a slightly lower interface friction angle of 22.0° compared with the smooth concrete-HDPE interfaces (Group D1). Additionally, the friction angle of the medium sand (MS)-HDPE interface is slightly higher than that of the FS-HDPE interface. It is worth noting that the shear resistance of soil-HDPE interfaces is heavily affected by the particle morphology and particle size of the soil. Owing to the fine particles and lubricity of water, the friction angle of the clay-HDPE interface is far less than sand-HDPE interfaces. Similarly, the friction angles of silty sand (SS)-HDPE interfaces and Ottawa sand (OS)-HDPE interfaces are also close to the result of the smooth concrete-HDPE interface.

The results from previous studies mentioned above are summarized in Table 5. There’s no denying that the comparisons reveal some key differences between concrete-HDPE and HDPEs that interact with other geotechnical materials.

**Table 5:** Summary of similar tests for the selected HDPE-sand/soil interfaces.

Samples	Sample size (mm)	Normal Stress (kPa)	Peak Shear Strength		Residual Shear Strength		Reference
			Friction Angle	Adhesion (kPa)	Friction Angle	Adhesion (kPa)	
FS-HDPE	300×300	22, 37, 53, 68	22.0°	-	17.3	-	Vangla and Gali [11]
MS-HDPE			22.2°	-	21.9	-	
MS-HDPE	100×100	49, 98.1, 196.2	19.7°	0	17.9	0	Hsieh [50]
	300×300		24.2°	0	18.9	0	
SS-HDPE	40×40	5, 12, 20, 30	21.0	2.5	18.4	2.8	Jogi [51]
SS-HDPE	100×100	5, 12, 20, 30	21.4~23.7	1.77~3.10	20.0~21.9	1.81~3.10	Fleming [47]
OS-HDPE			21.0	2.52	18.4	2.95	
OS-HDPE	60×60	0~50	22	2.76	-	-	Izgin and Wasti [52]
Clay-HDPE	400×600	50, 100, 200	10.8°	0	8.7	0	Feng and Lu [53]

### Concrete-HDPE interface with cementing action

In actual engineering, the mechanical stabilization of bonding joints between concrete and HDPE is also important. However, limited studies have been conducted on shear behaviors of concrete-HDPE interface with cementing action. Similarity, extensive research has been conducted on cemented paste backfill (CPB)-rock interface, CPB-CPB interface, and cemented concrete-rock interface [54–56]. The main observation is that, for the same interfaces and same normal stress, the cemented bonding condition can improve the shear strength. Additionally, curing time of cement also has a significant impact on mechanical behaviors. This will directly lead to the increase of friction angle of the concrete-geomaterial interface. Further research is needed to provide an understanding of the behavior of concrete-HDPE interface with cementing action.

## Conclusion

With the composite shaft lining as the research background, the interface behavior of the concrete-HDPE has been studied. A series of direct shear tests have been carried out on interfaces considering the effects of surface roughness and normal pressure. The results of this study will contribute to a more reasonable design of the composite shaft lining. Significant results and findings of this work are given below.

1. The shear behaviors of the investigated joints present diverse patterns. Most of the patterns obtained in this study are plowing wear, localized plastic deformation, and dilatancy, which are based on direct shear tests in the case of matching planes.
2. A notably linear relationship between the peak shear strength and normal stress can be found in all groups. Higher normal stress is associated with higher peak stress, and the interface friction angles increase with increasing JRC values.
3. The results of shear strength shows that the slope of the asperity mainly controlled the peak shear strength of the concrete-HDPE interface, and the Patton model cannot accurately predict the peak strength.

## Supporting information

S1 File (RAR)
